# Analysis of inflammatory cytokine and TLR expression levels in Type 2 Diabetes with complications

**DOI:** 10.1038/s41598-017-07230-8

**Published:** 2017-08-09

**Authors:** Saket Gupta, Ashwini Maratha, Jakub Siednienko, Anandan Natarajan, Thusitha Gajanayake, Shu Hoashi, Sinéad Miggin

**Affiliations:** 1 Immune Signalling laboratory, Institute of Immunology, Department of Biology, Maynooth University, Maynooth, Ireland; 2 Midlands Regional Hospital, Mullingar, Co. Westmeath Ireland; 3 School of Medicine, University College Dublin, Dublin, Ireland; 4 Hirszfeld Institute of Immunology and Experimental Therapy, Polish Academy of Sciences, Rudolfa Weigla, Wrocław, Poland

## Abstract

The pathogenesis and complications of type 2 diabetes (T2DM) are closely linked with defective glucose metabolism, obesity, cardiovascular disease and an inability to mount an effective immune response to certain pathogenic organisms. Perturbations in key innate immune receptors known as Toll-like receptors (TLRs) and inflammatory mediators such as IL-6, TNFα and IL-1β have been linked with T2DM. Herein, we sought to establish whether patients with T2DM and underlying complications exhibit perturbations in cytokine and TLR expression. Serum cytokine and mRNA levels of cytokines/TLRs in monocytes (M) and neutrophils (N) were measured in a cohort of 112 diabetic patients: good glycaemic control without complications (GC), good glycaemic control with complications (GCC), poor glycaemic control without complications (PC) and poor glycaemic control with complications (PCC) and compared them with 34 non-diabetic volunteers (NGT). Serum cytokine levels were normal in all study participants. In the GC group, cytokine and TLR gene expression were enhanced compared to NGT. In contrast, suppressed cytokine and TLR gene expression were evident in PC, GCC & PCC groups when compared to the GC. In conclusion, whereas serum pro-inflammatory cytokine levels are unaltered in T2DM patients, differences in inflammatory gene profiles exist among the T2DM patient groups.

## Introduction

Type 2 diabetes (T2DM) is the most prevalent human metabolic disease and its effects are now the leading cause of human morbidity and mortality^1^. T2DM, characterized by hyperglycaemia, is strongly associated with both macrovascular (such as coronary artery disease, peripheral arterial disease, and stroke) and microvascular complications (such as diabetic retinopathy, nephropathy and neuropathy)^2^. Studies have suggested that low-grade inflammation, characterized by pro-inflammatory cytokine production, is involved in the pathogenic processes causing T2DM^3,4^. Further, excessive production of these cytokines in T2DM has been associated with the development of microvascular and macrovascular complications and may predict the development of diabetes^3^. It has been shown that inflammatory processes in the pancreatic islets, adipose tissue (AT), liver and muscle provoke insulin resistance (IR) and β-cell dysfunction and may therefore antedate the diagnosis of diabetes^5^. The AT is a major source of cytokines such as TNF-α, IL-6 and monocyte chemoattractant protein-1 (MCP-1)^6–8^. Further, blood monocytes and neutrophils are sentinel cells that drive inflammation and concomitant pro-inflammatory cytokine production *in vivo*^3,9^. Many studies have also shown that the gene expression and phenotypic profiles of monocytes and neutrophils are altered in response to glucose and in a T2DM milieu^9–13^. Given this, we opted to study whether alterations in inflammatory gene expression occurred in both monocytes and neutrophils in the context of T2DM.

Toll-like receptors (TLRs) are a variety of pathogen pattern recognition receptors that serve to detect pathogen associated molecular patterns (PAMPs) present on pathogens including bacteria and viruses and to detect danger-associated molecular patterns (DAMPs)^14,15^. Studies have also shown that TLR2 and TLR4 expression levels are elevated in patients with T2DM^16,17^. In fact, TLR4 has been shown to play an important role in the pathogenesis of atherosclerosis, diet-induced obesity, and the related insulin resistance^18^. In the present study, we opted to explore the expression levels of key cytokines, chemokines and all TLRs in T2DM subjects and to explore whether alterations are linked with micro- and macrovascular complications, and duration of diabetes.

## Results

### Participant characteristics

The role of various risk factors for the development and progression of diabetes have been demonstrated by several epidemiologic studies^19,20^. These factors include type and duration of diabetes, age, gender, glycaemic control, hypertension, body mass index, smoking, serum lipids and presence of microalbuminuria; all these risk factors were examined in the current study (Table 1). Females were 67.6% in NGT and 42.9% in T2DM group. The median age was higher in T2DM subjects compared to NGT controls (p < 0.001) (Table 1). The duration of diabetes was calculated for all groups and as it reflects the course of disease, the groups with complications and poor glycaemic control group had longer duration of diabetes compared to good glycaemic control group (p < 0.001). The profile for smoking was similar in both NGT and T2DM groups: 20.5% were smokers in NGT compared to 20.5% in T2DM groups, and 30.7% were ex-smoker in NGT as compared to 43.5% in T2DM. There were significant differences in the BMI and WHR among the groups (p < 0.01) wherein the T2DM subjects had higher BMI and WHR compared to NGT controls. The median systolic BP (but not diastolic) was significantly raised in GC, GCC and PCC (p < 0.05). The fasting glucose was higher in all T2DM subjects compared to NGT controls (p < 0.05). All T2DM subjects had raised total cholesterol and triglycerides, and low HDL compared to NGT controls (p < 0.05). LDL levels were lower in T2DM subjects compared to NGT; this may be attributed to lipid lowering agents.

**Table 1:** Characteristics of Study Participants.

Characteristics	NGT	GC	GCC	PC	PCC	p value
Number	34	27	32	21	32	
Sex (M/F)	11/23	12/15	20/12	13/8	19/13	0.068
Age (years)	53 [47–59]	62 [51–71]	65 [57–73]	56 [50–60]	60 [55–68]	<0.001
Duration of DM (months)	‒	24 [12–72]	96 [30–168]	108 [60–132]	132[84–180]	<0.001
Smoker Y/N	7/24	3/21	6/26	5/16	9/22	0.659
Ex smoker Y/N	8/26	8/19	14/18	4/17	8/24	0.271
BMI (kg/m^2^)	26 [24–30]	32 [29–38]	31 [28–35]	35 [31–37]	34 [29–37]	<0.001
Waist (cm)	91 [82–100]	106 [99–115]	108 [99–115]	110 [99–120]	113 [102–122]	<0.001
WHR	0.92 [0.84–0.96]	0.96 [0.90–1.04]	1.03 [0.95–1.06]	1.04 [0.93–1.18]	1.02 [0.97–1.08]	<0.001
SBP (mmHg)	129 [120–137]	145 [131–156]	142 [131–154]	130 [120–145]	147 [124–158]	0.005
DBP (mmHg)	80 [77–85]	80 [71–85]	78 [73–84]	78 [73–88]	77 [68–86]	0.808
Fasting glucose (mM)	4.8 [4.7–5.1]	6.5 [5.8–7.2]	7.6 [6.1–8.2]	10.9[8.3–12.9]	10.6 [7.9–14.6]	
HbA1c (%)	5.6 [5.4–5.7]	6.4 [6.0–6.9]	6.9 [6.4–7.4]	10.2 [9.2–11]	9.6 [8.9–10.8]	
CRP	0.10 [0.06–0.25]	0.20 [0.09–0.30]	0.18 [0.06–0.50]	0.15 [0.06–0.60]	0.20 [0.10–0.40]	
Total cholesterol (mM)	5.9 [5.1–6.6]	3.9 [3.5–4.7]	3.7 [3.1–4.3]	4.2 [3.7–4.8]	3.9 [3.5–4.7]	
LDL cholesterol (mM)	3.6 [2.8–4.3]	2.2 [1.9–2.9]	1.8 [1.5–2.4]	2.3 [1.8–2.9]	2.0 [1.5–2.7]	
HDL cholesterol (mM)	1.6 [1.3–1.9]	1.3 [1.0–1.5]	1.1 [0.9–1.3]	1.1 [1.0–1.3]	1.1 [0.9–1.2]	
Triglycerides (mM)	0.9 [0.7–1.2]	1.5 [1.1–2.0]	1.4 [0.8–2.0]	1.5 [0.8–1.9]	1.8 [1.2–2.5]	

Data are expressed as median [Inter quartile range]. BMI, Body Mass Index, SBP, Systolic Blood Pressure, DBP, Diastolic Blood Pressure, WHR, Waist-to-hip ratio Data are expressed as median [Inter quartile range]. p value corresponds to the differences between groups. HbA1c, glycosylated haemoglobin, HDL, high-density lipoprotein, LDL, low-density lipoprotein, CRP, C-reactive protein.

Previous epidemiological studies have demonstrated increased concentrations of CRP in patients with T2DM^21^. In the present study, we aimed to evaluate patients with ‘steady state’ T2DM. Therefore, individuals with acute and chronic illness and a concomitant significant elevation in CRP levels (raised CRP levels multiples higher than baseline associated with conditions such as common cold, skin infections, urinary tract infections, joint inflammation, active autoimmune conditions such as Crohn’s disease, conditions with arterial inflammation such as polymyalgia rheumatism and malignancy) were excluded from the study. The aforementioned exclusion criteria was adopted since very high CRP (indicative of illness in both controls and diabetic patients) may act as a confounder in evaluating the baseline ‘steady state’ of pro- and anti- inflammatory forces. Notably, the inclusion and exclusion criteria that were adopted in the study were thorough and strictly adhered to; patients that met with our inclusion and exclusion criteria with mildly raised CRP were not excluded in this study. In the present study, a significant difference in CRP levels was not observed between different groups (Table 1).

A detailed drug history was obtained for all patients and it was observed that a higher percentage of patients in PC (52.4%) and PCC (71.9%) were administered insulin compared to GC (7.4%) and GCC (28.1%) groups (Table 2). In all groups, most T2DM subjects were administered lipid lowering agents: GC (85.2%), GCC (93.8%), PC (61.9%) and PCC (87.5%) and also administered angiotensin converting enzyme (ACE) inhibitor and angiotensin receptor blocker (ARB) in view of HTN or micro/macro albuminuria: GC (74.1%), GCC (87.5%), PC (76.2%), and PCC (81.3%) (Table 2).

**Table 2:** Baseline characteristics: medications and HTN in different subgroups.

Characteristics	NGT	GC	GCC	PC	PCC
n	34	27	32	21	32
Lipid lowering Agents	—	23	30	13	28
Aspirin	—	20	25	13	29
Metformin	—	22	26	18	19
Sulphonylurea	—	7	14	16	19
PPAR γ agonists	—	0	3	1	1
GLP 1 analogs	—	2	1	2	1
DPP IV inhibitors	—	0	3	2	2
Insulin	—	2	9	11	23
ACE inhibitors	—	16	20	8	19
ARB	—	4	8	8	7
HTN	—	17	6	7	20

PPAR γ agonists, Peroxisome proliferator-activated receptor gamma agonists, GLP-1 analogs, Glucagon-like peptide-1 analogs, DPP IV inhibitors, Inhibitors of dipeptidyl peptidase 4, ACE inhibitor, Angiotensin-converting-enzyme inhibitor, ARB, angiotensin receptor blockers, HTN, Hypertension.

All PCC patients had one or more microvascular complications (retinopathy, neuropathy and nephropathy). Additionally, 53.1% (17 patients) of these had one or more macrovascular complications. In GCC group, 56.3% (18 patients) had one or more microvascular complications, 34.4% (11 patients) had both macro & microvascular complications and 9.4% (3 patients) had only macrovascular complications. Among microvascular complications, retinopathy was higher in PCC patients (40.6%) compared to GCC patients (18.6%). No significant difference was observed in neuropathy in between two subgroups. 15.6% of PCC patients had diabetic foot disease compared to 6.3% GCC patients. Of the PCC patients, 68.8% had nephropathy compared to 56.3% in GCC subgroup. The proportion of patients having CKD was higher in PCC subgroup than GCC subgroup (CKD stage 3; 45.5% vs. 27.7%, CKD stage 4; 9% vs. 0%) (Table 3).

**Table 3:** CKD staging and diabetic nephropathy of GCC and PCC study participants.

Characteristics	GCC	PCC
n	32	32
Nephropathy	18	22
MicroAlb	14	18
MacroAlb	4	4
CKD	—	—
Stage 1	—	—
Stage 2	8	7
Stage 3	5	10
Stage 4	—	2
Stage 5	—	—

CKD, Chronic Kidney Disease, MicroAlb, Microalbuminuria, MacroAlb, Microalbuminuria.

### Levels of cytokines in sera from NGT and T2DM patients

**Figure 1:**
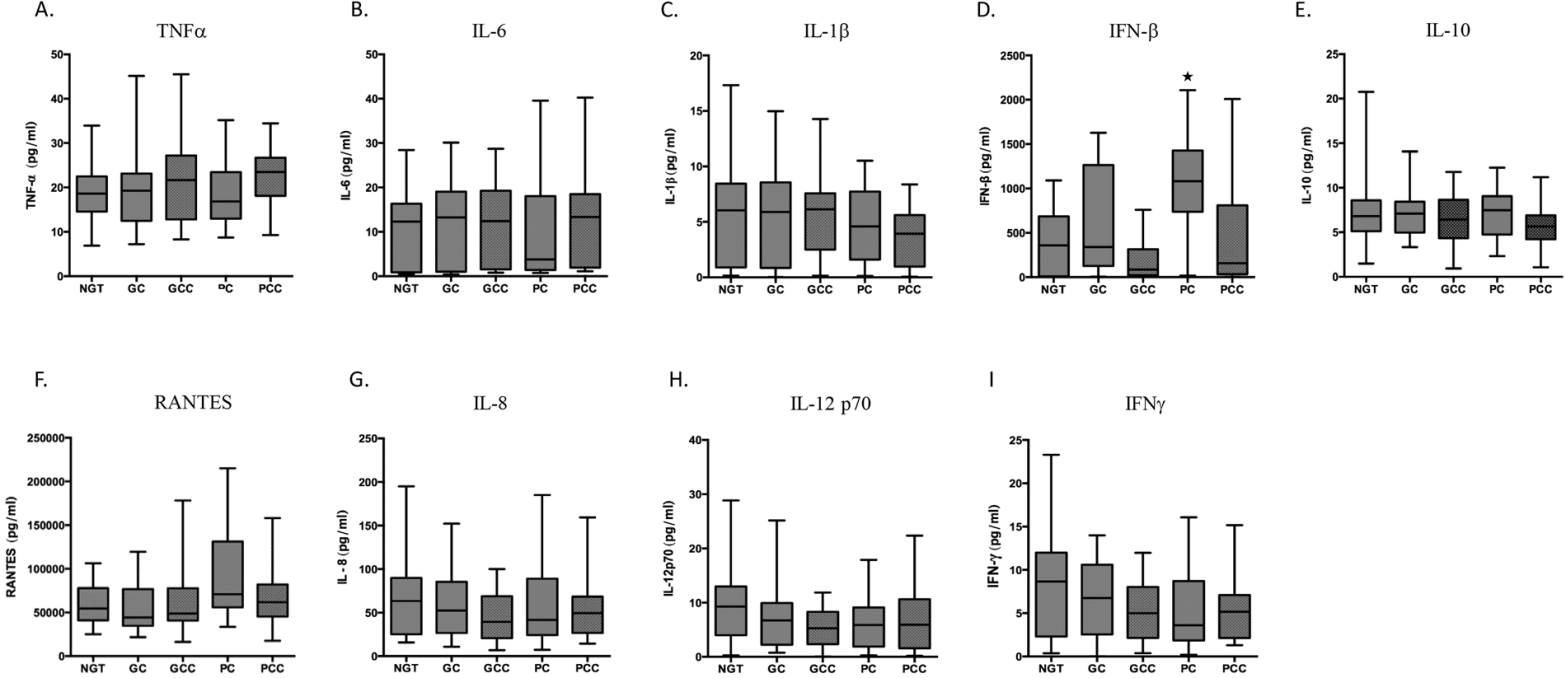
Levels of cytokines in sera from NGT and T2DM patients. TNF-α (**A**), IL-6 (**B**), IL-1β (**C**), IFN-β (**D**), IL-10 (**E**), Rantes (**F**), IL-8 (G) IL-12 (**H**) and IFNγ **(I**) levels in NGT (n = 34), GC (n = 27), GCC (n = 32), PC (n = 21) and PCC (n = 32) patient groups. *p < 0.05, **p < 0.01, ***p < 0.001 vs. NGT.

Following the analysis of cytokines/chemokine levels in serum, there was no statistical significant difference in serum TNFα, IL-6, and IL-1β levels among the different groups (Fig. 1A–C). The median values of serum TNFα and IL-6 were comparable in GC, GCC and PCC patients compared to NGT. While comparable levels of serum IL-1β were observed in GC and GCC, levels were decreased in PC and PCC, when compared with NGT. Serum IFN-β was significantly increased in PC and comparable in GC group, when compared to NGT control (Fig. 1D) and decreased in the GCC and PCC group (p < 0.05). However, following adjustment for covariates, serum IFN-β levels became statistically insignificant. Following the analysis of serum IL-10, RANTES, IL-8, IL-12 p70 and IFNγ, there was no statistical significant difference in serum levels among the different groups (Fig. 1E–I). Whereas a trend towards increased serum IL-10 was observed in GC and PC patients, a marginal trend towards suppressed IL-10 was observed in GCC and PCC patients, compared with NGT (Fig. 1E). Following assessment of RANTES, whereas a trend towards increased levels in PC and PCC patients was observed when compared with NGT control group, levels were comparable in GC, GCC and NGT (Fig. 1F). Whereas a trend towards decreased IL-8 was observed in GCC, PC and PCC, when compared with NGT, levels were comparable in GC and NGT (Fig. 1G). A trend towards decreased levels of both IL-12 and IFNγ was detected in the serum taken from T2DM subjects when compared to the NGT control group.

Next, multifactorial ANOVA analysis of the serum cytokines measured in the present study was undertaken after adjustment for age, sex, WHR, BMI, duration of diabetes, creatinine and medications (insulin, sulfonylurea, metformin, GLP-1 analogues, DPP IV inhibitors, aspirin and statins). No significant difference in cytokine levels among the study groups was shown and there was no correlation between serum cytokine levels and BMI in T2DM subjects.

### Gene expression of TNFα, IL-6, IL-1β, IFN-β and Rantes in monocytes and neutrophils from NGT and T2DM patients

**Figure 2:**
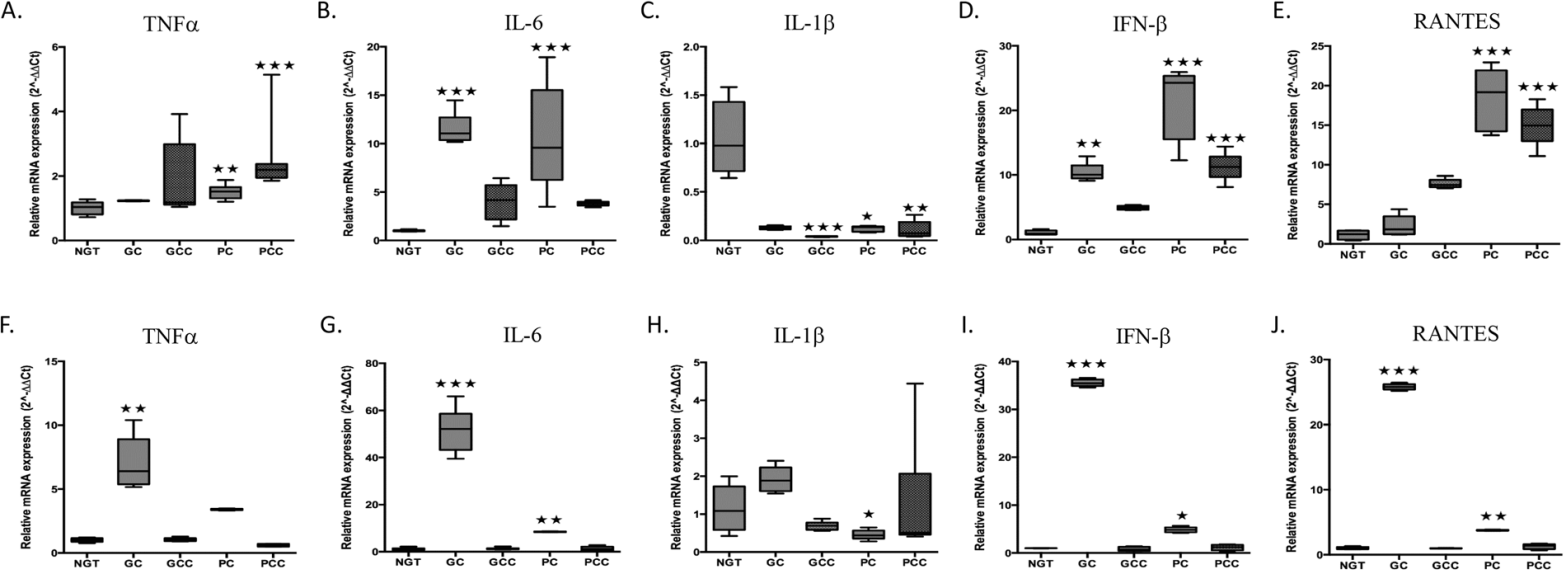
Gene expression of TNFα, IL-6, IL-1β, IFN-β and Rantes in monocytes and neutrophils from NGT and T2DM patients. Analysis of TNFα (**A**,**F**), IL-6 (**B**,**G**), IL-1β (**C**,**H**), IFN-β (**D**,**I**) and RANTES (**E**,**J**) gene expression in monocytes (**A**–**E**) and neutrophils (**F**–**J**) from NGT and T2DM patients (n = 8 per group). Top and bottom horizontal lines of the boxplots indicate 25th and 75^th^ percentiles respectively; lines within the box indicate median values. *p < 0.05, **p < 0.01, ***p < 0.001 vs NGT.

The transcriptional regulation of cytokines involved in regulating the inflammatory response were analysed using a quantitative PCR approach wherein the 2^−(ΔΔCt)^ method was used to analyse the relative changes in gene expression^22^. As expected, monocytes expressed mRNA encoding TNFα, IL-6, IL-1β, IFN-β and RANTES. A significant increase in TNFα mRNA expression was observed in the monocytes obtained from PC and PCC subjects when compared to NGT (Fig. 2A). A significant increase in IL-6 mRNA expression was observed in the monocytes obtained from GC and PC subjects when compared to NGT (Fig. 2B). A significant decrease in IL-1β mRNA expression was observed in the monocytes obtained from all T2D subjects when compared to NGT (Fig. 2C). Interestingly, IFN-β mRNA expression was increased in monocytes from GC, PC and PCC subjects when compared to NGT (Fig. 2D). Similarly, the highest levels of RANTES mRNA was observed in monocytes obtained from PC and PCC subjects (Fig. 2E). Levels of IL-6 (p < 0.05), IL-1β (p < 0.05), IFN-β (p < 0.001) and RANTES (p < 0.001) mRNA expression in monocytes remained significant among different groups following multifactorial ANOVA after adjustment for age, sex, WHR, BMI, duration of diabetes, creatinine and medications (insulin, sulfonylurea, metformin, GLP-1 analogues, DPP IV inhibitors, aspirin and statins). Additionally, a statistically significant correlation was found between IL-1β mRNA in monocytes and BMI in T2DM subjects (Spearman’s *r* = 0.41, p < 0.05). In summary, the highest level of IL-6 mRNA was observed in GC group, whereas highest level of IFNβ mRNA and Rantes mRNA were observed in the PC group, when compared to NGT. In contrast, IL-1β mRNA remained suppressed in the T2DM groups, compared to NGT.

In neutrophils, a significant increase in TNFα mRNA expression was observed in GC subjects when compared to NGT (Fig. 2F). A significant increase in IL-6 mRNA expression was observed in neutrophils obtained from GC and PC subjects when compared to NGT (Fig. 2G). A significant decrease in IL-1β mRNA expression was observed in the neutrophils obtained from PC subjects when compared to NGT (Fig. 2H). IFN-β and RANTES mRNA was increased in neutrophils from GC and PC subjects when compared to NGT (Fig. 2I,J). TNFα (p < 0.001), IL-6 (p < 0.001), IFN-β (p < 0.001) and RANTES (p < 0.001) mRNA expression levels remained significant among different groups following multifactorial ANOVA after adjustment for age, sex, WHR, BMI, duration of diabetes, creatinine and medications (insulin, sulfonylurea, metformin, GLP-1 analogues, DPP IV inhibitors, aspirin and statins). A correlation between cytokine mRNA levels and BMI was not evident in neutrophils. Collectively, our data suggests that whereas comparable TNFα, IL-6, IFN-β and RANTES mRNA expression levels were evident among the GCC, PCC and NGT groups, TNFα, IL-6, IFN-β and RANTES mRNA expression levels were highest in neutrophils obtained from GC subjects, when compared to NGT controls. In contrast, IL-1β mRNA expression levels were not elevated among the T2DM subject groups.

### Gene expression of TLR1-10 in monocytes and neutrophils from NGT and T2DM patients

**Figure 3:**
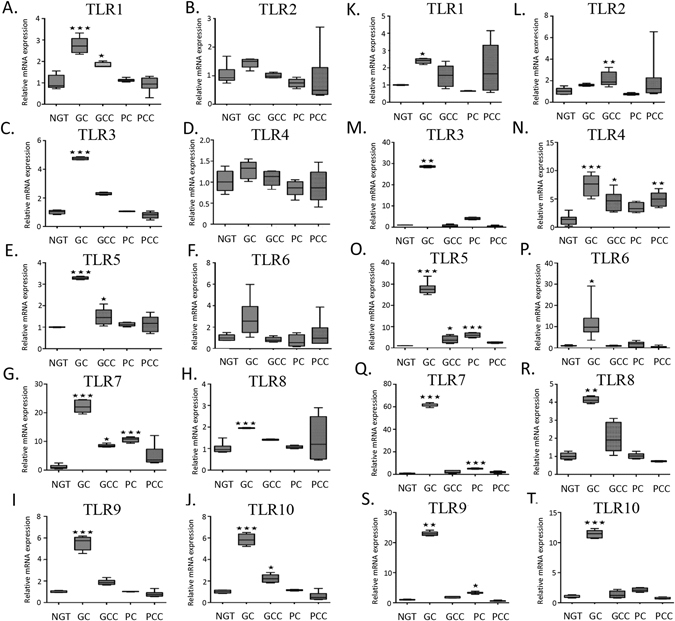
Gene expression of TLR1-10 in monocytes and neutrophils from NGT and T2DM patients. Analysis of TLR1 (**A**,**K**), TLR2 (**B**,**L**), TLR3 (**C**,**M**), TLR4 (**D**,**N**), TLR5 (**E**,**O**), TLR6 (**F**,**P**), TLR7 (**G**,Q), TLR8 (**H**,**R**), TLR9 (**I**,**S**), TLR10 (**J**,**T**) gene expression in monocytes (**A**–**J**) and neutrophils (**K**–**T**) from NGT and T2DM patients (n = 8 per group). Top and bottom horizontal lines of the boxplots indicate 25th and 75^th^ percentiles respectively; lines within the box indicate median values. *p < 0.05, **p < 0.01, ***p < 0.001 vs NGT.

In monocytes, TLR1, 3, 5 and 7–10 mRNA levels were elevated in GC subjects when compared to NGT; levels of TLR1, 3, 5, 7, 9, and 10 remained statistically significant following adjustment for age, sex, WHR, BMI, duration of diabetes, creatinine and medications (insulin, sulfonylurea, metformin, GLP-1 analogues, DPP IV inhibitors, aspirin and statins) using multifactorial ANOVA (Fig. 3A–J). In contrast, TLR1, 5, 7 & 10 levels were elevated in monocytes obtained from GCC subjects compared to NGT (p < 0.05) (Fig. 3A–J). In monocytes obtained from PC subjects, whereas TLR7 expression was enhanced, expression of all other TLRs was normal when compared to NGT. Interestingly, in PCC monocytes, expression of all TLRs examined was comparable to NGT.

In neutrophils, TLRs1, 3–10 mRNA levels were elevated in GC subjects when compared to NGT; levels remained statistically significant following adjustment for covariates as mentioned previously (Fig. 3K–T). Whereas TLR2, 4–5 levels were elevated in GCC when compared to NGT, only TLR4 mRNA expression was elevated in the PCC subjects (Fig. 3K–T). TLR5, 7 and 9, but not other TLRs, were elevated in neutrophils obtained from PC subjects. A correlation between TLRs mRNA levels in either neutrophils or monocytes, and BMI was not evident in the T2DM subjects.

### Correlation between fasting glucose levels, insulin and inflammation

In the GC group, there was a significant correlation between fasting glucose and monocyte IL-1β (r = 0.738; p = 0.04) and TLR8 (r = 0.94, p = 0.001), and neutrophil RANTES (r = −0.742, p = 0.04) mRNAs. In the GCC group, there was a significant correlation between fasting glucose and monocyte RANTES (r = 0.747; p = 0.03) and neutrophil TLR5 (r = −0.730, p = 0.04) mRNAs. In the PCC group, there was a significant correlation between fasting glucose and serum RANTES (r = 0.403; p = 0.02) and monocyte IL-1β mRNA (r = 0.857, p = 0.02).

In the GC group, there was a significant correlation between duration of diabetes and monocyte RANTES (r = −0.715, p = 0.05) and TLR8 (r = 0.741, p = 0.04) mRNA, and neutrophil TLR2 mRNA (r = −0.838, p = 0.01). In the GCC, there was no correlation. In the PC group, there was a correlation between neutrophil TLR8 (r = 0.771, p = 0.03) and TLR9 (r = 0.747, p = 0.04) mRNAs. In the PCC group, there was a correlation between duration of diabetes and neutrophil TLR1 (r = −0.730, p = 0.04), TLR3 (r = 0.766, p = 0.03) and TLR5 (r = 0.843, p = 0.01) mRNAs.

## Discussion

The main objective of this study was to investigate the role of inflammatory biomarkers in the pathophysiology of T2DM. To the best of our knowledge, this is the first study that has sought to evaluate serum cytokine levels and cytokines/TLRs gene expression in both monocytes and neutrophils among T2DM cohorts.

In this study, we showed that in neutrophils, the highest levels of the proinflammatory cytokines, TNFα, IL-6 and IFN-β mRNA were present in the GC subjects when compared to NGT control. Similarly, in monocytes, IL-6 and IFN-β mRNA levels were elevated in GC subjects, when compared to NGT. Our results correlate with previous studies which showed that TNFα mRNA and IL-6 levels were elevated in T2DM subjects^23,24^. Chemokines, such as IL-8 and RANTES play a critical role in driving and inflammatory milieu since they mediate the arrival of inflammatory cells to the site of both acute and chronic inflammation^25^. Herein, our study demonstrated that RANTES mRNA expression was elevated in neutrophils (but not monocytes) obtained from the GC subjects. Our study correlates, in part, with previous studies, which demonstrate that serum RANTES levels are increased in T2DM subjects^25,26^. Intriguingly, our study showed that while RANTES mRNA levels were increased in some of the T2DM subjects, levels of RANTES in serum were comparable among the different groups.

Our study also demonstrated that TNFα, IL-6, IFN-β and RANTES mRNA and serum levels were not significantly elevated in GCC subjects when compared to NGT. In the PC subjects, while levels of IL-6, IFN-β and RANTES mRNA were raised in both monocytes and neutrophils, serum protein levels were not significantly different among the groups.

In the PCC subjects, while levels of IFN-β and Rantes mRNA were raised in monocytes, levels remained unchanged in neutrophils, when compared to NGT. Despite an increase in cytokine mRNA level, serum protein levels were not significantly different among the groups. Further TNF-α mRNA was significantly overexpressed in PC and PCC monocytes, but after adjustment for covariates, it became statistically insignificant.

Regarding IL-12, we observed low serum levels of IL-12 and IFN-γ in T2DM subjects, which was statistically insignificant. Our findings are in contrast to that previously reported by Tsiavou *et al*., who demonstrated low serum levels of IFN- γ and IL-12 in T2DM subjects^27^. In contrast, Wegner *et al*., demonstrated elevated IL-12 serum levels in T2DM subjects^28^. Regarding IL-10, an anti-inflammatory cytokine, our study demonstrated that IL-10 levels were comparable in T2DM and NGT subjects, however it remained statistically insignificant. This is in contrast to Yaghini *et al*., who previously showed that IL-10 levels were decreased in T2DM subjects^29^.

Contrasting with TLR2 and TLR4, there is a paucity of data exploring the role of other TLRs in T2DM. However, upregulated expression of TLR1–9 and TLR11–13 was shown in two murine models of obesity^30^. Given that TLRs are key enablers of the inflammatory response, serving to drive the production of inflammatory cytokines, we sought to explore the expression levels of TLR1-10 in monocytes and neutrophils obtained from T2DM and healthy participants. In monocytes, our study demonstrated that, in general, levels of TLR mRNA expression were enhanced in the monocytes obtained from GC subjects. Similarly, in neutrophils, expression of most TLR mRNAs was increased in GC subjects when compared to NGT. Correlating with our data generated from T2DM patients with good glycaemic control (GC), previous studies have demonstrated that the expression of TLR2 and TLR4 was increased in monocytes derived from patients with T2DM, mean duration 29 months^17^. Further, other studies demonstrated that TLR2 and TLR4 mRNA levels were elevated in PBMCs taken from T2DM patients^31^, though mean disease duration was not indicated^32^.

Interestingly, in our study, T2DM patients with mean disease duration >8 years, namely T2DM patients with poor glucose control, and subjects with complications showed a trend towards suppressed TLR mRNA expression when compared to GC subjects. In monocytes, whereas TLR7 levels were elevated in PC, levels of all TLRs were comparable to NGT among PCC subjects. Similarly, in neutrophils, GCC subjects moderately overexpress TLR4 & 5, whereas PC overexpressed TLR5, 7 & 9 compared to NGT. PCC subjects overexpressed only TLR4 in neutrophils, and no significant change in the expression of TLR3, 6, 8, 9 & 10 was noted in PCC compared to NGT. Further, after adjustment for age, sex, WHR, BMI, duration of diabetes, creatinine and medications using lognormal data with multifactorial ANOVA, TLR1, 3, 5, 6, 7, 9 and 10 mRNA levels remained statistically significant among the groups.

Taken together, these data suggest that TLR gene expression is elevated in T2DM patients with good glucose control when compared to the general population. However, in T2DM patients with good glucose control with complications, levels of TLRs are decreased when compared to individuals with similar glucose control without complications. Further, in T2DM patients with poor glucose control in the absence and presence of complications, levels of TLR gene expression are decreased when compared to T2DM patients with good glucose control. In fact, levels of TLR expression among patients with poor glucose control are more comparable to that observed in the general population. Given the apparent differences in the expression of TLRs among the T2DM groups, namely the enhanced TLR expression in patients with good glucose control versus the downregulation of TLRs in patients with poor glucose control and in patients with complications, it is plausible to speculate that decreased TLR expression in the latter groups may have occurred to curtail sustained TLR activation and concomitant inflammatory processes. Whether decreased TLR expression leads to compromised innate immune signalling mechanisms and concomitant predisposition to infections requires further investigation.

Our study has shown that IL-6 and IFN-β mRNA levels are elevated in monocytes and neutrophils from GC and PC and that TNFα mRNA levels are elevated in neutrophils from GC group, compared with NGT. Intriguingly, our study failed to show elevated serum TNFα, IL-6, IFNβ, IL-8, IL-10, IL-12 and IFNγ levels in T2DM subjects despite perturbations in cytokine/TLR mRNAs levels being evident in the monocytes and neutrophils taken from T2DM patients. In terms of TLR expression, studies have shown that metformin^33^, statins, PPAR-γ agonists^34^ and angiotensin receptor blockers^35^ lower the expression of TLR2 and TLR4 in T2DM patients. Further, previous studies have shown that insulin infusion significantly suppressed TLRs 1, 2, 4, 7, and 9 mRNA expressions in mononuclear cells obtained from T2DM individuals^18^. Notably, in the current study, a greater number of T2DM patients from the GCC, PC and PCC were prescribed insulin compared to the GC patient group. Given that our T2DM subjects were prescribed and were administering these medications, it is plausible to speculate that long term administration of these drugs, for example in the GCC, PC and PCC groups, may contribute to the impaired TLR expression among the cohort when compared to the GC group.

In conclusion, our study shows that while perturbations in the gene expression of cytokines are evident in patients with T2DM, serum levels of the cytokines examined remain comparable between individuals with and without T2DM. Further, our study also suggests that levels of TLRs, receptors intimately linked with the inflammatory process, are perturbed in patients with good glucose control in the absence of complications. However, levels of TLR mRNA expression decrease as the disease duration progresses over time. Whether this perturbation is the result of disease process itself, the prescribed medications, or whether patients with perturbed inflammatory markers are more susceptible to developing diabetes and associated microvascular and macrovascular complications, remains to be investigated. However, the consistent and significantly suppressed cytokine and TLR gene expression in patients with poor glycaemic control and with complications suggests a “burnt out” disease state. Landmark clinical trials (UKPDS, Steno 2, ADVANCE) have already shown that intensive glycaemic control leads to a reduction in morbidity and mortality over time^36–40^. This study raises the potential role of early aggressive glucose lowering and cardiovascular risk factor management in reversing the peak inflammation associated with the early stages of T2DM and thus reducing the risk of cardiovascular and microvascular complications later in life. Further investigation is required to examine this hypothesis.

## Research Design and Methods

### Study subjects and design

Healthy control participants were screened for diabetes using the standard oral glucose tolerance test (American Diabetes Association (ADA)). Healthy control participants with abnormal blood results (diabetes or pre-diabetes) and individuals with a history of chronic illness and/or individuals taking medication were excluded from the NGT group. T2DM participants had established diagnosis according to the ADA criteria. All participants were >18 years old and were not pregnant. Exclusion criteria included the following: Evidence of current infection (white cell count >11 or CRP >normal range), current treatment with antibiotics, neutropenia (a leucocyte count of less than 2000 per cubic millimetre), pregnancy or breast-feeding, non diabetic renal disease or liver disease (aspartate aminotransferase or alanine aminotransferase of more than three times the upper limit of the normal range), ongoing or previous cancer and the use of oral/inhaled glucocorticoids, immunosuppressive treatment or immunodeficiency.

Based on these criteria, 146 participants were recruited from a regional hospital. Healthy volunteers without diabetes acted as the control group (normal glucose tolerance, NGT; n = 34) and 112 T2DM patients (mean duration 95 months) with four different profiles were recruited: T2DM with good glycemic control (HbA1c <7.5%) and no macrovascular complications (heart disease, transient ischaemic stroke (TIA)/stroke or peripheral arterial disease (PAD)) and microvascular complications (retinopathy, neuropathy or nephropathy) GC, n = 27), T2DM with good glycemic control and complications (GCC, n = 32), T2DM with poor glycemic control (HbA1c >7.5%) without complications (PC, n = 21), and T2DM with poor glycaemic control and complications (PCC, n = 32). In case of the healthy volunteers, each participant met the inclusion and exclusion criteria for the study. Each healthy control participant was screened for diabetes and prediabetes using the standard oral glucose tolerance test^41^. Participants with abnormal results were excluded from the study. Personal and medical data was obtained by patient interview, by using hospital medical notes, and from using hospital laboratory test results. The demographic information was obtained from research participants and their weight, height, blood pressure & waist and hip circumferences were determined. BMI was calculated as body weight (in kilograms) divided by body height (in meters) squared. Waist-to-hip ratio (WHR) was calculated as waist divided by hip circumference. Informed consent was obtained from all participants. The protocol was approved by the Midlands Research Ethics Committee, Health Service Executive, Ireland, and by the Ethical Review Board, Maynooth University, Maynooth, Ireland and all experiments were performed in accordance with relevant guidelines and regulations.

### Biochemical Analysis

Routinely, fasting blood samples were collected from consenting T2DM patients and healthy control subjects in the morning between 8–10 am to minimise the impact of diurnal variation on the study. More specifically, about 40 ml peripheral blood was collected from each participant including healthy volunteers and was used to measure various biochemical parameters and cytokines. A haemoglobin analyser HA-8160 (Menarini Pharmaceuticals, Ireland) was used to measure HbA1C (HPLC Chromatography Method) and Advia analysers were used to measure the whole blood counts. Lipid profile, urea, creatinine, sodium, potassium, asparate aminotransferase, alanine aminotransferase, bilirubin, alkaline phosphatase, gamma glutamyl transferase, CRP, ferritin, coagulation screen, thyroid profile and plasma glucose were measured using a Roche Modular 1800 analyser. An early morning urine sample for ACR measurement was also collected at the same time.

### Cytokine measurements

For cytokine analysis, the blood was placed on ice and then centrifuged (3000 rpm, at 4 °C) within 1 hour of venesection. Serum was collected and stored at −80 °C. Serum was used for measuring TNFα, IL-6, IL-1β, IL-10, IL-8, IL-12p70 and IFN-γ using a 96-Well MULTI-ARRAY 7-Multiplex Assay (Meso Scale Discovery). Serum was used for measuring IFN-β and Rantes using single-plex 96-well plates (Meso Scale Discovery).

### Mononuclear cell isolation and TLR mRNA quantification

Peripheral Blood Mononuclear Cells (PBMC) were isolated from heparinized peripheral blood by density gradient centrifugation. Human CD14 positive monocytes were isolated from PBMCs using the EasySep™ Human CD14 Positive Selection Cocktail, and neutrophils were isolated using the EasySep™ Human Neutrophil Enrichment Kit by negative selection. Initially, to ensure the robustness of the isolation procedure, a number of samples were analysed by flow cytometry to establish and confirm the purity of the monocyte and neutrophil population post selection. Next, total RNA was extracted from monocytes and neutrophils using the RNeasy isolation kit (Qiagen). RNA (1 μg per 25 μl reaction) was converted to first strand cDNA and stored at −20 °C^42^. Real-time PCR quantification was performed with DyNAmoHS SYBR Green kit (Finnzymes) using the OPTICON® system (MJ Research). For the amplification of TLRs & cytokines, in house-designed primers were used (Supplemental Table S1) using the housekeeping gene hypoxanthine phosphoribosyl transferase (HPRT) as a reference point^42^.

### Statistical analysis

Data are expressed as median (interquartile range) and represented as box-and-whiskers plots. The dark midline in the box represents the distribution’s median value. The top and bottom edges of the box respectively represent the 75th and 25th percentile values. The top and bottom of the vertical lines, the whisker respectively represent the upper and lower maximum value. χ2 test or Fisher exact test (as appropriate) were used to compare proportions. The Mann–Whitney test was used in case of non-normally distributed parameters to compare median between two groups. Kruskal-Wallis test was used for multiple inter-group comparisons in case of parameters that did not show normal distribution. If a significant difference was found in inter-group comparisons, post hoc multiple comparison analysis with the Dunns multiple comparison test was performed. Data were transformed to log normal format and multifactorial ANOVA was applied to evaluate the effect of variables (age, sex, BMI, WHR and duration of diabetes) on different inflammatory markers. Correlations between values were examined by calculating Spearman correlation coefficients. All the statistical analysis was performed using the Prism 6.0 computer program (GraphPad, La Jolla, CA) and p value less than 0.05 was considered significant.

## Electronic supplementary material

Supplemental Figure S1
