# Author Correction: Analysis of inflammatory cytokine and TLR expression levels in Type 2 Diabetes with complications

**DOI:** 10.1038/s41598-018-23475-3

**Published:** 2018-04-05

**Authors:** Saket Gupta, Ashwini Maratha, Jakub Siednienko, Anandan Natarajan, Thusitha Gajanayake, Shu Hoashi, Sinéad Miggin

**Affiliations:** 1 Immune Signalling laboratory, Institute of Immunology, Department of Biology, Maynooth University, Maynooth, Ireland; 2 Midlands Regional Hospital, Mullingar, Co., Westmeath, Ireland; 3 School of Medicine, University College Dublin, Dublin, Ireland; 4 Hirszfeld Institute of Immunology and Experimental Therapy, Polish Academy of Sciences, Rudolfa Weigla, Wrocław, Poland

Correction to: *Scientific Reports* 10.1038/s41598-017-07230-8, published online 09 August 2017

This Article incorrectly indicates that Saket Gupta, Ashwini Maratha, Shu Hoashi and Sinéad Miggin jointly supervised this work, rather than Shu Hoashi and Sinéad Miggin only.

In addition, Saket Gupta and Ashwini Maratha contributed equally to this paper.

