# Supplementary material for: Analysis of inflammatory cytokine and TLR expression levels in Type 2 Diabetes with complications
Source: Sci Rep. 2017 Aug 9;7:7633. doi: 10.1038/s41598-017-07230-8 (PMC5550417; doi:10.1038/s41598-017-07230-8)
Supplement: Supplementary file 1 — Supplemental Figure S1 [file 41598_2017_7230_MOESM1_ESM.pdf]

Analysis of inflammatory cytokine and TLR expression levels in Type 2 Diabetes with complications

Saket Gupta[2*], Ashwini Maratha[1*], Jakub Siednienko[1, 4], Anandan Natarajan[2], Thusitha Gajanayake[1], Shu Hoashi[2,3*] and Sinéad Miggin[1*]

[1] Immune Signalling laboratory, Institute of Immunology, Department of Biology, Maynooth University, Maynooth, Ireland;

[2] Midlands Regional Hospital, Mullingar, Co. Westmeath, Ireland;

[3] School of Medicine, University College Dublin, Dublin, Ireland

[4] Hirszfeld Institute of Immunology and Experimental Therapy, Polish Academy of Sciences, Rudolfa Weigla, Wrocław, Poland.

joint senior authorship

Gupta et al., Supplemental Table S1: Primers used for the amplification of human genes (TLR, cytokine/chemokine) and HPRT-housekeeping gene.

Human Gene	Gene accession number	Forward primer sequence (5'-3')	Reverse primer sequence (5'-3')	Predicted fragment size (bp)	Annealing Temperature (T_m, ^oC)
HPRT	NM_000194.2	AGCTTGCTGGTGAAAAGGAC	TTATAGTCAAGGGCATATCC	104	60
TLR1	NM_003263.3	TATTCCTCCTGTTGATATTGCTGCT	TAAATGGTGAACTGCGACCCGAAG	135	60
TLR2	NM_003264.3	ACCTGTCCAACAACAGGATCACCT	TGTTCAAGACTGCCCAGGGAAGAA	139	60
TLR3	NM_003265.2	AAGAACTCACAGGCCAGGAATGGA	AAGAGGCTGGAATGGTGAAGGAGA	182	60
TLR4	NM_138554.3	GCCGAAAGGTGATTGTTGTGGTGT	TACCAGCACGACTGCTCAGAAACT	108	60
TLR5	NM_003268	GTTGCAACTTGCCTGGGAAACTGA	AGCCTGTTGGAGTTGAGGCTTAGT	162	60
TLR6	NM_006068.2	TGAGGTTAGCCTGCCAGTTAGAGA	TTTGGGAAAGCAGAGTGGAGAGGA	126	60
TLR7	NM_016562	TATTCCCACGAACACCACGAACCT	GCAGCCTCTTGATGCACATGTTGT	164	60
TLR8	NM_138636	TGTCTCAGAGGCTGCAATGTAGGT	AGGCTCGCATGGCTTACATGAGTA	136	60
TLR9	NM_017442	CCACAACAACATCCACAGCCAAGT	TGGGACAAGTCCAGCCAGATCAAA	162	60
IL-6	NM_000600.3	AGCCACTCACCTCTTCAGAACGAA	CAGTGCCTCTTTGCTGCTTTCACA	121	60

TNFα	NM_000594.2	CACCACTTCGAAACCTGGGA	CACTTCACTGTGCAGGCCAC	115	60
IFN-β	NM_002176.2	AACTGCAACCTTTCGAAGCC	TGTCGCCTACTACCTGTTGTGC	123	60
CCL5	NM_002985.2	TGCCTGTTTCTGCTTGCTCTTGTC	TGTGGTAGAATCTGGGCCCTTCAA	92	60
IL-1β	NM_000576	ACAGATGAAGTGCTCCTTCCA	GTCGGAGATTCGTAGCTGGAT	73	60
